# ARE LIFE ACCOMPLISHMENTS RELATED TO WELL-BEING: FINDINGS FROM THE HEALTH AND RETIREMENT STUDY (HRS)?

**DOI:** 10.1093/geroni/igae098.3499

**Published:** 2024-12-31

**Authors:** Marina Larkina, Jacqui Smith

**Affiliations:** University of Michigan, Ann Arbor, Michigan, United States; University of Michigan, Ann Arbor, Michigan, United States

## Abstract

Butler (1963) proposed that engaging in a whole life review is especially beneficial for older adults because it promotes a sense of self-worth. Previous research highlights links between reminiscing and psychological well-being, however, life review narratives are rarely collected in the context of large, population-based aging studies. This study uses data from the HRS Life History Mail Survey (2017, 2019) to 1) examine factors that predict older adults (N=12,815; Mage= 68 years; range 50-101) willingness to share their life’s most important accomplishments (up to three); and 2) establish relations between content and complexity (content variety) of reported accomplishments and participants’ well-being. Participants who reported their accomplishments (59%), compared to those who didn’t, were more likely to be better educated, currently married, wealthier, have better memory and fewer depressive symptoms. Importantly, they also were more likely to perceive their life-as-a-whole as either completely or very satisfied (OR=1.315, CI=1.214-1.425, p<.001). Multinomial analysis of content and complexity of reported accomplishments revealed that people with higher life satisfaction were more likely to report relationship-related accomplishments (47%; family, friendship, or caring for others) compared to those who were most proud of their societal achievement (35%; education, work, military services), adjusted RRR=1.207, CI=1.061-1.372, p<.005. People, who wrote about personal qualities (e.g., their faith, health) did not differ from other two groups. The complexity of reported accomplishments was not associated with life satisfaction but was related to respondents’ education and memory. Our findings support the proposal that reviewing one’s life accomplishments enhances well-being in later life.

